# Predictors to Use Mobile Apps for Monitoring COVID-19 Symptoms and Contact Tracing: Survey Among Dutch Citizens

**DOI:** 10.2196/28416

**Published:** 2021-12-20

**Authors:** Stephanie Jansen-Kosterink, Marian Hurmuz, Marjolein den Ouden, Lex van Velsen

**Affiliations:** 1 eHealth Department Roessingh Research and Development Enschede Netherlands; 2 Biomedical Signals and Systems Group University of Twente Enschede Netherlands; 3 Technology, Health & Care Saxion University of Applied Sciences Enschede Netherlands

**Keywords:** COVID-19, eHealth, mHealth, contact tracing, symptom management, intention to use

## Abstract

**Background:**

eHealth apps have been recognized as a valuable tool to reduce COVID-19’s effective reproduction number. The factors that determine the acceptance of COVID-19 apps remain unknown. The exception here is privacy.

**Objective:**

The aim of this article was to identify antecedents of acceptance of (1) a mobile app for COVID-19 symptom recognition and monitoring and (2) a mobile app for contact tracing, both by means of an online survey among Dutch citizens.

**Methods:**

Next to the demographics, the online survey contained questions focusing on perceived health, fear of COVID-19, and intention to use. We used snowball sampling via posts on social media and personal connections. To identify antecedents of the model for acceptance of the 2 mobile apps, we conducted multiple linear regression analyses.

**Results:**

In total, 238 Dutch adults completed the survey; 59.2% (n=141) of the responders were female and the average age was 45.6 years (SD 17.4 years). For the symptom app, the final model included the predictors age, attitude toward technology, and fear of COVID-19. The model had an r2 of 0.141. The final model for the tracing app included the same predictors and had an r2 of 0.156. The main reason to use both mobile apps was to control the spread of the COVID-19 virus. Concerns about privacy was mentioned as the main reason to not use the mobile apps.

**Conclusions:**

Age, attitude toward technology, and fear of COVID-19 are important predictors of the acceptance of COVID-19 mobile apps for symptom recognition and monitoring and for contact tracing. These predictors should be taken into account during the development and implementation of these mobile apps to secure acceptance.

## Introduction

It is spring 2020 and the COVID-19 pandemic has the world in its grip. Infection with COVID-19 can lead to a simple cold or no symptoms at all, while it can also rapidly develop into a life-threatening disease, especially for patients with existing cardiovascular problems, obesity, or diabetes [1]. To hamper the spread of COVID-19 and to manage the intensive care unit capacity, many countries have applied a lockdown strategy for their citizens [2]. In order to control the spread of COVID-19 after a lockdown, and to minimize the effective reproduction number of the disease, several measures can be applied, of which social distancing, combined with aggressive case finding and isolation, seems to be the most effective [3].

eHealth apps have been recognized as a valuable tool for supporting symptom recognition and monitoring [4], for contact tracing [5], and ultimately, for reducing COVID-19’s effective reproduction number by means of timely intervention. In short, a contact tracing app would record a citizen’s contacts with other people via Bluetooth technology and, in the case of a COVID-19 infection, will warn the persons that the index patient recently had contact with so that they can apply self-isolation and be attentive for any COVID-19 symptoms. However, for such apps to be effective, high uptake among the population is necessary. For the case of a tracing app, it has been estimated that 56% of a country’s population should use the app to suppress the epidemic [6]. It is therefore crucial that the design of these apps and the implementation strategies that accompany them take the factors that affect acceptance into account.

The factors that determine acceptance of COVID-19 apps are largely unknown [7]. The exception here is privacy. Since the initial plan of governments to implement these technologies, a fierce public debate erupted on whether or not large-scale tracing of contacts for this goal is an unacceptable breach of privacy or not. While the issue of privacy has been recognized as an important antecedent of acceptance of mobile health apps [8], the unique and disturbing situation that the COVID-19 pandemic places us in makes it difficult to apply existing models and frameworks for eHealth acceptance. In May 2020 the Dutch government wanted to develop and implement 2 mobile apps to prevent the spread of the COVID-19 virus and support Dutch municipal health services. The aim of this article was to identify antecedents of acceptance of (1) a mobile app for COVID-19 symptom recognition and monitoring, and (2) a mobile app for contact tracing, both by means of an online survey among Dutch citizens.

## Methods

### Overview

To identify antecedents of acceptance of a mobile app for COVID-19 symptoms recognition and monitoring (hereafter: symptom app), and a mobile app for contact tracing (hereafter: tracing app), an online survey was developed, tested, and distributed among Dutch citizens. This study did not require formal ethical approval (as ruled by CMO Oost Nederland, file number: 2020-6628). At the beginning of the survey, participants were asked for consent to use their data for research purposes.

### Survey

#### Design

The online survey (Multimedia Appendix 1) consisted of 4 parts. The first part included questions on demographics, the second part contained questions related to perceived health, the third part consisted of questions related to the fear of a COVID-19 infection, and the final part included questions to assess the intention to use the 2 suggested mobile apps. In April 2020, the Dutch government announced plans to develop and implement 2 mobile apps for preventing the spread of the COVID-19 virus. However, the exact design of these apps remained unknown at this time. Therefore, we introduced both mobile apps in the survey via a short description of their general aim. We pretested the survey with 14 Dutch citizens to improve legibility.

#### Demographics

We assessed gender, age, smartphone use, educational level (student, primary school, secondary school, high school, bachelor’s degree/university/PhD), work status (unemployed and searching for work, not able to work due to illness, volunteer work, part-time work, full-time work, retired, student), income level (below-average wages, average wages, above-average wages), and living status (living alone, living together, other). We assessed the participants’ attitude toward technology using the Personal Innovativeness in the Domain of Information Technology scale by Agarwal and Prasad [9], consisting of 4 statements and accompanied by a 5-point Likert scale (ranging from 1 [strongly disagree] to 5 [strongly agree]). Finally, we also asked whether participants were (once) infected with COVID-19. The answer options for this question were: Yes, In doubt, or No.

#### Perceived Health

To assess perceived health, we asked participants to complete 3 questions. These questions were used previously to assess perceived health among Dutch citizens [10]. These questions/statements were (1) How would you describe your health?; (2) How concerned are you about your health?; and (3) I am ill more often than other people of the same age and sex. These were accompanied by a 5-point Likert scale ranging from 1 (bad, not concerned, and totally disagree, respectively) to 5 (excellent, very concerned, and totally agree, respectively).

#### Fear of COVID-19

The participants’ fear of a COVID-19 infection was assessed by means of 4 questions related to this topic:

- Have you been concerned about the outbreak of the COVID-19 virus in recent weeks?: 5-point Likert scale, ranging from 1 (not at all concerned) to 5 (extremely concerned)
- How often did you think of the outbreak of the COVID-19 virus in recent weeks?: 5-point Likert scale, ranging from 1 (never) to 5 (always)
- How afraid were you of the outbreak of the COVID-19 virus in recent weeks?: 5-point Likert scale, ranging from 1 (not afraid at all) to 5 (very afraid)
- How afraid are you of getting sick from the COVID-19 virus?: 5-point Likert scale, ranging from 1 (not afraid at all) to 5 (very afraid)

#### Intention to Use

Finally, participants were asked to rate their intention to use the 2 mobile apps: (1) a symptom app and (2) a tracing app. The statements for the construct intention to use were based on van Velsen et al [11]. All 3 questions were accompanied by a 5-point Likert scale ranging from 1 (strongly disagree) to 5 (strongly agree). Next to these closed questions, respondents were also asked what the main reasons were to “use” and “not to use” the mobile apps.

### Survey Distribution

Distribution of the survey (via QualtricsXM) started on April 15, 2020. Participants were eligible if they were 18 years of age or older. We used a snowball sampling via posts on social media (LinkedIn, Twitter, and Facebook) and personal connections. Next to this, we recruited participants via a Dutch panel of older adults that indicated they were interested in participating in research on the topic of eHealth. The survey was closed on April 30, 2020. Due to the method of recruitment, a response rate could not be calculated.

### Analyses

Data were analyzed using SPSS (version 19; IBM). Descriptive statistics were performed for all outcomes. Cronbach α values were calculated to assess internal consistency for attitude toward technology, perceived health, fear of COVID-19, and intention to use. Next, survey scores were interpreted for these factors as being negative (score 1 or 2), neutral (score of 3), or positive (score 4 or 5). Via a paired (2-tailed) *t* test, the difference in intention to use score between both mobile apps was tested. To identify antecedents of acceptance of (1) a symptom app and (2) a tracing app, we conducted multiple linear regression analyses (backward model analyses). The intention to use each app was used as the dependent variable. The independent variables were selected based on Pearson correlation coefficients. Demographic characteristics and factors that (borderline) significant correlated (Pearson correlation cut-off level *P*≤.10) with the dependent variable “intention to use” were included in the multiple linear regression analyses. For the paired *t* test and regression analyses, the level of significance was set at *P*<.05. For the final models the r^2^ was calculated, which indicates the percentage of the variance in the dependent variable that the independent variables explain collectively. To support the quantitative results, the responses on the 2 open questions were sorted and counted by the first author and discussed with the second author, taking an inductive approach. Disagreements were discussed until unanimous agreement was reached.

## Results

### Composition of Survey Participants

In total, 238 Dutch citizens completed the survey. Fifteen responders only completed the intention to use survey of a tracing app as this app was presented first and these responders stopped with the survey after these questions; 59.2% (141/238) of the responders were female and the average age was 45.6 years (SD 17.4 years). Only 2.1% (5/238) of responders did not own a smartphone and 74.8% (178/238) claimed that they carried their smartphone with them for most of the day. The average age of our sample is higher than the average age of the Dutch population. There was also an overrepresentation of female participants and participants with a high education level [12]. Compared with the statistics of 2018, in the current sample, there is an overrepresentation of participants owning a smartphone and compared with the statistics of 2020 there is an underrepresentation of participants who are unemployed [12]. The internal consistency of the Attitude Toward Technology scale was good (Cronbach α=.85). Most responders (176/238, 73.9%) had a moderate attitude toward technology. Only 3/238 responders (1.3%) claimed to be infected with COVID-19. All demographic characteristics are presented in Table 1.

**Table 1:** Responders’ demographics (n=238).

Demographics		Values
**Gender, n (%)**		
	Male	97 (40.8)
	Female	141 (59.2)
Age (years), mean (SD)		45.6 (17.4)
**Smartphone, n (%)**		
	Yes	233 (97.9)
	No	5 (2.1)
**Carry smartphone with you?, n (%)**		
	Always	178 (74.8)
	Sometimes	55 (23.1)
	Never	5 (2.1)
**Education level, n (%)**		
	Student	16 (6.7)
	Primary school	2 (0.8)
	Secondary school	14 (5.9)
	High school	57 (23.9)
	Bachelor’s degree/university/PhD	149 (62.6)
**Work status, n (%)**		
	Unemployed and searching for work	3 (1.3)
	Not able to work due to illness	8 (3.4)
	Volunteer work	2 (0.8)
	Part-time work	75 (31.5)
	Full-time work	81 (34.0)
	Retired	43 (18.1)
	Student	25 (10.5)
**Income level, n (%)**		
	Below-average wages	76 (31.9)
	Average wages	93 (39.1)
	Above-average wages	69 (29.0)
**Living status, n (%)**		
	Living alone	34 (14.3)
	Living together	191 (80.3)
	Other	13 (5.5)
**Attitude toward technology,^a^ mean (SD)**		3.2 (0.78)
	Low (1-2)	3 (1.3)
	Moderate (3)	176 (73.9)
	High (4-5)	59 (24.8)
**COVID-19 infection, n (%)**		
	Yes	3 (1.3)
	In doubt	44 (18.5)
	No	191 (80.3)

^a^Measured with a scale from 1 (low) to 5 (high).

### Fear of a COVID-19 Infection

The internal consistency of the 4 items in this scale was acceptable to good (Cronbach α=.78). The mean score on this topic was 3.3 (SD 0.68). The majority of the responder’s opinion on this topic was neutral (192/238, 80.7%) and 16% (38/238) of the responders were afraid for a COVID-19 infection. Only a few responders (8/238, 3.4%) were not afraid (Table 2).

**Table 2:** Descriptive statistics and internal consistency of scales.

Scale	Number of items	Cronbach α	Mean (SD)	Positive, n (%)	Neutral, n (%)	Negative, n (%)
Fear of COVID-19 (n=238)	4	.78	3.3 (0.68)	38 (16.0)	192 (80.7)	8 (3.4)
Perceived health (n=238)	3	.69	3.8 (0.68)	139 (58.4)	97 (40.8)	2 (0.8)
Intention to use the symptom app (n=223)	3	.96	3.38 (1.07)	101 (45.3)	101 (45.3)	21 (9.4)
Intention to use the tracing app (n=238)	3	.96	3.27 (1.14)	98 (41.2)	108 (45.4)	32 (13.4)

### Perceived Health

For the 3 items to assess the perceived health of the responders the internal consistence was acceptable (Cronbach α=.69). The mean score on this scale was 3.8 (SD 0.68). Most respondents were positive about their health (139/238, 58.4%).

### Intention to Use

The intention to use was assessed for the symptom app and the tracing app. For both scales, internal consistency was excellent (Cronbach α for the symptom app=.96 and Cronbach α for the tracing app=.96). For both apps, the majority’s intention to use was neutral (Table 2). However, an additional paired *t* test indicated that there was a significant difference in the scores on intention to use for the symptom app (mean 3.38 [SD 1.07]; n=223) and the tracing app (mean 3.27 [SD 1.13]; n=223; *t*_222_=–2.598 and *P*=.01), indicating that the responders were more willing to use a mobile app for COVID-19 symptom recognition and monitoring compared with a mobile app for contact tracing.

### Correlations

The intention to use the symptom app was related to income level (r=0.132, *P*=.05), attitude toward technology (r=0.220, *P*<.001), and fear of COVID-19 (r=–0.291, *P*<.001). The intention to use the tracing app was related to age (r=0.135, *P*=.04), attitude toward technology (r=0.223, *P*<.001), and fear of COVID-19 (r=–0.303, *P*<.001). Based on these outcomes, the independent variables within the linear regression analysis were age, income level, attitude toward technology, fear of COVID-19, and perceived health. Table 3 provides an overview of the correlations between all demographics and factors, and the intention to use.

**Table 3:** Outcome Pearson correlation.

Variables	Intention to use the symptom app (n=223)	Intention to use the tracing app (n=238)
Gender	r=–0.056 *P*=.41	r=–0.147 *P*=.23
Age	r=0.126 *P*=.06	r=0.135^a^ *P*=.03
Education level	r=0.21 *P*=.76	r=0.018 *P*=.79
Work status	r=0.072 *P*=.28	r=0.033 *P*=.62
Income level	r=–0.132^a^ *P*=.05	r=0.124 *P*=.06
Living status	r=0.083 *P*=.22	r=0.060 *P*=.35
Attitude toward technology	r=0.220^a^ *P*<.001	r=0.223^a^ *P*<.001
Fear of COVID-19	r=–0.291^a^ *P*<.001	r=–0.303^a^ *P*<.001
Perceived health	r=–0.088 *P*=.19	r=–0.119 *P*=.07

^a^Correlation is significant at the .05 level (2-tailed).

### Linear Regression

A multiple linear regression analysis was conducted to predict the intention to use a symptom app based on age, income level, attitude toward technology, fear of COVID-19, and perceived health. The final model included the predictors attitude toward technology, fear of COVID-19, and age (*F*_3,3_=12.012; *P*<.001). The model has an r^2^ of 0.141. It contains 3 factors that affect the intention to use, but only 2 of them are significant predictors:

- Fear of COVID-19: β=–.272, *t*_3_=4.305, *P*<.001
- Attitude toward technology: β=.222, *t*_3_=3.532, *P*=.001
- Age: β=.107, *t*_3_=1.691, *P*=.09 (not significant)

Another multiple linear regression analysis was conducted to predict the intention to use a tracing app based on age, income level, attitude toward technology, fear of COVID-19, and perceived health. The final model included the predictors attitude toward technology, fear of COVID-19, and age (*F*_3,3_=14.333; *P*<.001). The model has an r^2^ of 0.155. Intention to use is predicted by:

- Fear of COVID-19: β=.286, *t*_3_=4.742, *P*<.001
- Attitude toward technology: β=.230, *t*_3_=3.815, *P*<.001
- Age: β=.128, *t*_3_=2.104, *P*<.05

### Main Reason to Use the Mobile Apps

An overview of all reasons the responders brought forth for using both mobile apps is presented in Tables 4 and 5. The main reason (33/116, 28.4) for responders to use the symptom app was to control the spread of the COVID-19 virus. In addition, respondents were willing to use this mobile app to monitor own complaints (22/116, 19.0%) and to gain more insight into the spread and symptoms of the COVID-19 virus (19/116, 16.4%).

The main reason to use a tracing app was also to control the spread of the COVID-19 virus (45/147, 30.6%). Next to this, respondents were willing to use this mobile app to gain more insight into the spread and symptoms of the COVID-19 virus (34/147, 23.1%) and for one’s own health (19/147, 12.9%).

**Table 4:** Overview of the main reasons to use the symptom app (n=116).

Reasons	Value, n (%)
To control the spread of the COVID-19 virus in general	33 (28.4)
To monitor own complaints	22 (19.0)
More insight into the spread and symptoms of COVID-19	19 (16.4)
To control the spread of the COVID-19 virus for oneself	15 (12.9)
For one’s own health	12 (10.3)
For safety	7 (6.0)
For society	5 (4.3)
To protect the frail population	2 (1.7)
Out of fear	1 (0.9)

**Table 5:** Overview of the main reasons to use the tracing app (n=147).

Reasons	Value, n (%)
To control the spread of the COVID-19 virus in general	45 (30.6)
More insight into the spread and symptoms of COVID-19	34 (23.1)
For one’s own health	19 (12.9)
For safety	17 (11.6)
To control the spread of the COVID-19 virus for oneself	15 (10.2)
For society	9 (6.1)
To protect the frail population	6 (4.1)
Out of fear	2 (1.4)

### Main Reason Not to Use the Mobile Apps

An overview of the reasons to not use the mobile apps is presented in Tables 6 and 7. For both mobile apps, privacy was mentioned as the main reason (symptom app=56.6% [64/113] and tracing app=64.8% [92/142]) to not use the mobile apps. Other reasons for not using the mobile apps were the expected usefulness of the app (symptom app=23.9% [27/113] and tracing app=13.4% [19/142]) and a fear of becoming over aware of the situation and its potential consequences, leading to unnecessary stress (symptom app=8.0% [9/113] and tracing app=11.3% [16/142]).

**Table 6:** Overview of the main reasons not to use the symptom app (n=113).

Reason	Value, n (%)
Privacy/not willing to share information with government	64 (56.6)
Doubting usefulness	27 (23.9)
Over awareness/stress	9 (8.0)
Doubting ease of use	5 (4.4)
Doubting security	5 (4.4)
No (compatible) phone	2 (1.8)
The fear the use of the app will be forced by government	1 (0.9)

**Table 7:** Overview of the main reasons not to use the tracing app (n=142).

Reason	Value, n (%)
Privacy/not willing to share information with government	92 (64.8)
Doubting usefulness	19 (13.4)
Over awareness/stress	16 (11.3)
No (compatible) phone	6 (4.2)
Doubting security	3 (2.1)
Doubting ease of use	3 (2.1)
The fear the use of the app will be forces by government	3 (2.1)

## Discussion

The aim of this paper was to identify antecedents of acceptance of (1) a mobile app for COVID-19 symptom recognition and monitoring, and (2) a mobile app for contact tracing among Dutch citizens by means of an online survey.

### Principal Results

Our main finding is that for both mobile apps age, attitude toward technology, and fear of COVID-19 are antecedents of acceptance. A large group of the Dutch citizens (101/223, 45.3%) is willing to use a mobile app for COVID-19 symptom recognition and monitoring. The main reasons to use this mobile app are to (1) control the spread of COVID-19; (2) monitor their own complaints, and (3) gain more insight into the spread and symptoms of the COVID-19 virus. For the case of a mobile app for COVID-19 contact tracing, 41.2% (98/238) of the Dutch adults appear to be willing to use this mobile app. The main reasons for use are (1) to control the spread of the COVID-19 virus, (2) to gain more insight into the spread and symptoms of the COVID-19 virus, and (3) for their own health. Privacy, doubting the usefulness of the mobile app, and a fear of becoming over aware of the situation and its potential consequences, leading to unnecessary stress, are the main reasons to not use the mobile apps. Overall, Dutch citizens were more willing to use a mobile app for COVID-19 symptom recognition and monitoring compared with a mobile app for contact tracing.

### Comparison With Prior Work

It is difficult to relate our findings to the existing literature, as limited technology acceptance studies have focused on mobile apps to be used during a pandemic, and insights into factors that determine the acceptance of COVID-19–related mobile apps are lacking [7]. In general, age and attitude toward technology are widely acknowledged antecedents of acceptance. For age there is evidence that older age is associated with a lower level of acceptance of mobile apps [13]. Previous results also indicated that attitude toward technology is an important antecedent of acceptance of mobile apps [13,14]. The degree to which an individual is willing to try out any new mobile app is related to the intention to use [13]. Since this study, the mobile apps, announced by the Dutch Government in April 2020, have been developed and implemented. In a recent study by Bente et al [15], the contact tracing app (the CoronaMelder) was tested for usability, and was found easy to use. A comparable study was executed in Germany by Blom et al [16]. They analyzed the potential barriers for the large-scale adoption of the official contact tracing app that was introduced in Germany. The foremost barrier toward using the contact tracing app was the lack of willingness to correctly adopt the app. Besides, compared with the younger group (aged 18-59 years), the older age group (aged 60-77 years) was less likely to use a compatible smartphone. Therefore, access was also mentioned as barrier in this study [16]. Another cross-country survey study (participating countries: France, Germany, Italy, the United Kingdom, and the United States) on the acceptance of a contact tracing app is more optimistic [17], as the willingness to install the app was high among all 5 counties and across all subgroups of the population. In addition, this study concluded that epidemiological evidence shows that app-based contact tracing can suppress the spread of COVID-19 if a high enough proportion of the population uses the app [17].

Our results show that fear of COVID-19 is the most important COVID-19–related factor that predicts acceptance of mobile apps to deal with the COVID-19 pandemic. Because it is difficult to translate this fear into a technology design, this finding needs to be seen in a bigger picture. Public health campaigns during the COVID-19 epidemic will need to educate citizens about the dangers of COVID-19 (personally and for society as a whole), and should then offer downloading COVID-19 mobile apps as a personal strategy to deal with this fear. Next, the positive attitude toward technology that precedes a decision to download a COVID-19 app should be taken into consideration when using these innovations. The end-user population might be skewed toward those with interest in technology (traditionally these are younger, highly educated men [18]), which can create a use divide, and thus, a health divide in society. Measures should be installed to support those groups in society that are not, by nature, technically interested, such as having promotional stalls in the community and diverse channels of user support.

### Limitations

The following 4 limitations should be taken into account for this study. First, due to our recruitment method (snowball sampling via social media), our sample could have been affected by a selection bias. Our sample was mainly composed of participants with a high educational level and a moderate attitude toward technology. Therefore, our results are based on the views of a somewhat skewed sample of the Dutch population, which might reduce the generalizability of our findings. Second, for our analysis, the power of our sample was sufficient. However, a larger sample would improve the generalizability of our outcomes, as mainly Dutch citizens from the eastern part of the Netherlands (87.0% [207/238] of our sample) completed our survey. Third, in our survey the 2 mobile apps are introduced by means of a short description of their general aim. It is unclear if this description was sufficient for the responders to understand the purpose of both mobile apps. Our survey was distributed before the development of the CoronaMelder app in the Netherlands. The study by Bente et al [15] indicated that during this period there were many misconceptions concerning contact tracing among the Dutch population. It is likely that these short descriptions of the general aim of the 2 mobile apps were insufficient to take those misconceptions away. Fourth, the explained variance of both our models is relatively low. Normally, in studies such as these, this number is boosted by including the predictors perceived ease of use and perceived usefulness. However, including these 2 factors leads to little practical results, that is, concluding that the apps should be easy to use. By contrast, the identification of COVID-19–related factors remains an important extension of the existing technology acceptance models.

### Conclusions

Age, attitude toward technology, and fear of COVID-19 are important predictors of the acceptance of COVID-19 mobile apps for symptom recognition and monitoring and for contact tracing. These predictors should be taken into account during the development and implementation of these mobile apps to secure acceptance.

## Appendix

Multimedia Appendix 1 Survey questions and answer options in Dutch and English (D=demographic questions; C=fear of COVID-19 questions; H=perceived health questions; TAM-BI=behavioural intention).
