# Supplementary material for: Predictors to Use Mobile Apps for Monitoring COVID-19 Symptoms and Contact Tracing: Survey Among Dutch Citizens
Source: JMIR Form Res. 2021 Dec 20;5(12):e28416. doi: 10.2196/28416 (PMC8691407; doi:10.2196/28416)
Supplement: Multimedia Appendix 1 [file formative_v5i12e28416_app1.docx]

## Multimedia Appendix 1

Table 8. Survey questions and answer options in Dutch and English. (D= demographic questions / C= fear of COVID-19 questions / H= perceived health questions / TAM-BI= behavioural intention)

|  |  | Dutch |  | English |  |
| --- | --- | --- | --- | --- | --- |
|  |  |  |  |  |  |
| D | 1 | Wat is uw geslacht? | o Man  o Vrouw | What is your gender? | o Men  o Women |
| D | 2 | Wat is uw leeftijd? |  | What is your age? |  |
| D | 3 | Wat zijn de 4 cijfers van uw postcode? | | What are the 4 digits of your zip code? | |
| D | 4 | Heeft u een smartphone? | o Ja  o Nee | Do you have a smartphone? | o Yes  o No |
| D | 5 | Draagt u uw smartphone de hele dag bij u? | o Nooit  o Soms  o Altijd | Do you carry your smartphone with you all day? | o Never  o Sometimes  o Always |
| D | 6 | Wat is de hoogste opleiding die u heeft afgerond? | o Basisschool  o Lbo, mavo, vmbo  o Mbo, havo, vwo  o Hbo, wo  o Ik studeer nog | What is the highest level of education you have completed? | o Primary school  o vocation education  o vocational education  o higher education  o I am still studying |
| D | 7 | Welke van de volgende categorieën beschrijft uw werkstatus het best? | o Werkloos, op zoek naar werk  o Werkloos, niet op zoek naar werk  o Part-time werkzaam  o Full-time werkzaam  o Gepensioneerd  o Door ziekte niet de mogelijkheid om te werken  o Student  o Vrijwilligerswerk | Which of the following categories best describes your work status? | o Unemployed, looking for work  o Unemployed, not looking for work  o Working part-time  o Working full-time  o Retired  o Not able to work due to illness  o Student  o Volunteering |
| D | 8 | Wat is uw gemiddelde inkomen? (Modaal inkomen = €36.000 bruto per jaar) | o Beneden modaal  o Rond modaal  o Boven modaal | What is your average income? (Average income = € 36,000 gross per year) | o Below average  o Around average  o Above average |
| D | 9 | Bent u alleen of woont u samen? | o Alleenstaand  o Samenwonend  o Anders | Are you single or do you live together? | o Single  o Living together  o Otherwise |
| D | 10 | Hoe denkt u over nieuwe technologieën in het algemeen? Vink voor elke stelling het antwoord aan dat het beste bij u past. | | How do you feel about new technologies in general? For each statement, tick the answer that suits you best. | |
|  |  | a. Als ik hoor over een nieuwe technologie, kijk ik ernaar uit om dat uit te proberen | o Zeer mee oneens  o Mee oneens  o Neutraal  o Mee eens  o Zeer mee eens | a. When I hear about a new technology, I look forward to trying it out | o Strongly disagree  o I disagree  o Neutral  o Agree  o Strongly agree |
|  |  | b. Vergeleken met de mensen in mijn omgeving ben ik meestal een van de eersten die nieuwe technologieën uitprobeert | o Zeer mee oneens  o Mee oneens  o Neutraal  o Mee eens  o Zeer mee eens | b. Compared to the people around me, I'm usually one of the first to try out new technologies | o Strongly disagree  o I disagree  o Neutral  o Agree  o Strongly agree |
|  |  | c. In het algemeen aarzel ik om nieuwe technologieën uit te proberen | o Zeer mee oneens  o Mee oneens  o Neutraal  o Mee eens  o Zeer mee eens | c. In general, I hesitate to try out new technologies | o Strongly disagree  o I disagree  o Neutral  o Agree  o Strongly agree |
|  |  | d. Ik probeer graag nieuwe technologieën uit | o Zeer mee oneens  o Mee oneens  o Neutraal  o Mee eens  o Zeer mee eens | I like to try out new technologies | o Strongly disagree  o I disagree  o Neutral  o Agree  o Strongly agree |
| D | 11 | Bent u al besmet geraakt met het COVID-19 virus? | o Ja  o Nee  o Ik twijfel | Have you already been infected with the COVID-19 virus? | o Yes  o No  o I doubt |
| C | 12 | Was u de afgelopen weken bezorgd over de uitbraak van het COVID-19 virus? | o Helemaal niet bezorgd  o Een klein beetje bezorgd  o Nogal bezorgd  o Bezorgd  o Heel erg bezorgd | Have you been concerned about the outbreak of the COVID-19 virus in recent weeks? | o Not at all concerned  o Slightly concerned  o Somewhat concerned  o Moderately concerned  o Extremely concerned |
| C | 13 | Hoe vaak dacht u de afgelopen weken aan de uitbraak van het COVID-19 virus? | o Nooit  o Zelden  o Soms  o Vaak  o Altijd | How often did you think of the outbreak of the COVID-19 virus in recent weeks? | o Never  o Rarely  o Sometimes  o Often  o Always |
| C | 14 | Hoe bang was u de afgelopen weken voor de uitbraak van het COVID-19 virus? | o Helemaal niet bang  o Een klein beetje bang  o Nogal bang  o Bang  o Heel erg bang | How scared were you of the outbreak of the COVID-19 virus in recent weeks? | o Not afraid at all  o A little bit afraid  o Quite afraid  o Scared  o Very scared |
| C | 15 | Hoe bang bent u om ziek te worden van het COVID-19 virus? | o Helemaal niet bang  o Een klein beetje bang  o Nogal bang  o Bang  o Heel erg bang | How afraid are you of getting sick from the COVID-19 virus? | o Not afraid at all  o A little bit afraid  o Quite afraid  o Scared  o Very scared |
| H | 16 | Hoe zou u uw gezondheid omschrijven? | o Slecht  o Matig  o Goed  o Zeer goed  o Uitstekend | How would you describe your health? | o Bad  o Poor  o Good  o Very good  o Excellent |
| H | 17 | Hoe bezorgd bent u over uw gezondheid? | o Helemaal niet bezorgd  o Een klein beetje bezorgd  o Nogal bezorgd  o Bezorgd  o Heel erg bezorgd | How concerned are you about your health? | o Not at all concerned  o Slightly concerned  o Somewhat concerned  o Moderately concerned  o Extremely concerned |
| H | 18 | Ik ben vaker ziek dan andere mensen van dezelfde leeftijd en hetzelfde geslacht. | o Zeer mee oneens  o Mee oneens  o Neutraal  o Mee eens  o Zeer mee eens | I am sick more often than other people of the same age and gender. | o Strongly disagree  o I disagree  o Neutral  o Agree  o Strongly agree |
| TAM | 19 | Hieronder krijgt u stellingen die gaan over uw verwachting over de app waarmee in kaart wordt gebracht met wie een besmet persoon in contact is geweest. Vink voor elke stelling het antwoord aan wat het best aan uw verwachting voldoet. | | Below you will find statements about your expectation about the app to determine if an infected person has been contacted with others. For each statement, tick the answer that best meets your expectations. | |
| TAM-BI | | a. Ik ben van plan deze App te gebruiken zo vaak als nodig is. | o Zeer mee oneens  o Mee oneens  o Neutraal  o Mee eens  o Zeer mee eens | a. I plan to use this App as often as necessary. | o Strongly disagree  o I disagree  o Neutral  o Agree  o Strongly agree |
|  |  | b. Als deze App beschikbaar zou zijn voor mij, zou ik deze absoluut gebruiken. | o Zeer mee oneens  o Mee oneens  o Neutraal  o Mee eens  o Zeer mee eens | b. If this App were available to me, I would absolutely use it. | o Strongly disagree  o I disagree  o Neutral  o Agree  o Strongly agree |
|  |  | c. Ik hoop dat deze App beschikbaar komt voor mij. | o Zeer mee oneens  o Mee oneens  o Neutraal  o Mee eens  o Zeer mee eens | c. I hope this App becomes available to me. | o Strongly disagree  o I disagree  o Neutral  o Agree  o Strongly agree |
|  | 20 | Wat is voor u de belangrijkste reden om gebruik te maken van deze App? | | What is the main reason for you to use this App? | |
|  | 21 | Wat is voor u de belangrijkste reden om geen gebruik te maken van deze App? | | What is the main reason for you not to use this App? | |
| TAM | 22 | Hieronder krijgt u stellingen die gaan over uw verwachting over de app om symptomen van u, als eventuele corona patiënt, te volgen. Vink voor elke stelling het antwoord aan wat het best aan uw verwachting voldoet. | | Below you will find statements about your expectation about the app to track your symptoms as corona patient. For each statement, tick the answer that best meets your expectations. | |
| TAM-BI | | a. Ik ben van plan deze App te gebruiken zo vaak als nodig is. | o Zeer mee oneens  o Mee oneens  o Neutraal  o Mee eens  o Zeer mee eens | a. I plan to use this App as often as necessary. | o Strongly disagree  o I disagree  o Neutral  o Agree  o Strongly agree |
|  |  | b. Als deze App beschikbaar zou zijn voor mij, zou ik deze absoluut gebruiken. | o Zeer mee oneens  o Mee oneens  o Neutraal  o Mee eens  o Zeer mee eens | b. If this App were available to me, I would absolutely use it. | o Strongly disagree  o I disagree  o Neutral  o Agree  o Strongly agree |
|  |  | c. Ik hoop dat deze App beschikbaar komt voor mij. | o Zeer mee oneens  o Mee oneens  o Neutraal  o Mee eens  o Zeer mee eens | c. I hope this App becomes available to me. | o Strongly disagree  o I disagree  o Neutral  o Agree  o Strongly agree |
|  | 23 | Wat is voor u de belangrijkste reden om gebruik te maken van deze App? | | What is the main reason for you to use this App? | |
|  | 24 | Wat is voor u de belangrijkste reden om geen gebruik te maken van deze App? | | What is the main reason for you not to use this App? | |
